# High-Efficiency Removal of Copper and Nickel via Donnan Dialysis Using Fujifilm Cation-Exchange Membranes: Process Optimization Through Response Surface Methodology

**DOI:** 10.3390/membranes15120346

**Published:** 2025-11-21

**Authors:** Nabila Chaabani, Sana Ncib, Lasâad Dammak, Christian Larchet, Wided Bouguerra, Elimame Elaloui

**Affiliations:** 1 Laboratory of Materials Application to Water, Environment and Energy (LAM3E), Faculty of Sciences of Gafsa, University of Gafsa, Sidi Ahmed Zarroug University Campus, Gafsa 2112, Tunisia; nabila.chaabani95@gmail.com (N.C.); sana.ncib84@gmail.com (S.N.); limamealoui@gmail.com (E.E.); 2 Institut de Chimie et des Matériaux Paris Est, ICMPE UMR-CNRS 7182-UPEC, Université Paris-Est Créteil, 2 rue Henri Dunant, 94320 Thiais, France; larchet@u-pec.fr

**Keywords:** Donnan dialysis, Fujifilm cation-exchange membranes, copper, nickel, response surface methodology, central composite design

## Abstract

This study investigates the simultaneous removal of copper and nickel ions from aqueous solutions using Donnan dialysis (DD), with process optimization performed through Response Surface Methodology (RSM). An initial screening identified the most effective counter-ion, guiding the experimental design toward optimal ion removal. Comparative experiments using Fujifilm Type 1 and Type 2 cation-exchange membranes revealed the superior performance of Type 1 for both metals, as confirmed by FTIR-ATR spectroscopic analysis of membrane morphology. A comprehensive experimental campaign based on a Central Composite Design (CCD) evaluated the effects of copper, nickel, and sodium ion concentrations on removal efficiency. Under optimized conditions—80 mg/L Cu^2+^, 100 mg/L Ni^2+^, and 0.06 mol/L Na^+^—the Fujifilm Type 1 membrane achieved remarkable removal rates of 99.67 ± 0.21% for copper and 98.85 ± 0.34% for nickel. These values were experimentally validated (99.45 ± 0.18% and 98.71 ± 0.27%, *n* = 3), showing excellent agreement between predicted and observed data and confirming the robustness of the optimized Donnan dialysis process. Overall, the results highlight Donnan dialysis as a highly efficient and environmentally sustainable approach for the removal and recovery of heavy metals from aqueous solutions using Fujifilm cation-exchange membranes.

## 1. Introduction

Wastewater containing residual acids and anthropogenic heavy metals is generated across various industrial sectors, including metallurgy, electroplating, paper manufacturing, and mining [1]. Rapid industrialization in recent years has significantly increased the production of wastewater laden with toxic substances, contributing to widespread environmental pollution [2].

Heavy metals are naturally occurring inorganic elements distributed throughout the Earth’s crust [3]. When their concentrations exceed permissible limits, they are considered pollutants. Heavy metals are typically defined as elements with a density greater than 4 g/cm^3^, encompassing both metals and metalloids [4]. Industrial discharges, agricultural runoff, stormwater, mining activities, and untreated sewage contribute to heavy metal contamination in freshwater systems, posing serious health and ecological risks.

Among the commonly reported heavy metals, copper and nickel are extensively used in modern technological industries [5,6]. Copper ranks third in global usage after iron and aluminum and plays a key role in the transition to green energy [7,8]. It is widely employed in electroplating, batteries, pesticides, galvanized piping, and various alloys [9,10,11,12,13]. Chronic exposure to copper-contaminated drinking water can cause gastrointestinal distress, including stomach upset, cramps, and diarrhea. Nickel, commonly used in stainless steel production, batteries, catalysts, and electroplating [14], is also prevalent in water and wastewater sources. Elevated nickel levels pose significant health risks, including lung and kidney damage, skin dermatitis, and pulmonary fibrosis.

Due to their similar physicochemical properties, copper and nickel often co-occur in nature, exhibiting complete mutual solubility and similar densities [15]. They are fully miscible in both liquid and solid states and frequently intergrown in mineral deposits, complicating their separation through conventional beneficiation methods [16].

Heavy metals enter the human body either through direct consumption of contaminated water or indirectly via the food chain, ultimately impacting public health [17,18]. In developing countries, contaminated water is responsible for approximately 70–80% of illnesses [19,20], exacerbated by the lack of effective water treatment infrastructure and limited access to safe drinking water [21].

Various techniques are available for heavy metal removal, including adsorption [22,23], electrocoagulation–electroflocculation [24,25,26], polymer inclusion membrane [27], chemical precipitation [28], ultrafiltration and nanofiltration [29,30], electrodialysis[31,32], forward osmosis [33], membrane extraction [34], ion-exchange [35], and Donnan dialysis [36,37,38].

Membrane-based separation processes, such as Donnan dialysis (DD), offer operational and energy advantages due to their compact design, ease of use, low cost, environmental friendliness, and reduced energy consumption [39,40]. DD utilizes an ion-exchange membrane to separate a feed solution containing target ions from a receiver solution containing counter-ions [41,42].

Ion transport in DD is driven by an electrochemical potential difference resulting from ion concentration gradients across the membrane. The receiver solution typically contains salts (e.g., NaCl or KCl), strong acids (e.g., HCl or H_2_SO_4_), or strong bases (e.g., NaOH or KOH) at higher concentrations than the target ions in the feed solution. Counter-ions from the receiver migrate into the feed solution, displacing the target ions in the opposite direction until Donnan equilibrium is reached [43].

The reliability and selectivity of ion removal depend heavily on the performance of the ion-exchange membrane. Fujifilm membranes were selected for this study due to their chemical stability, high ion-exchange capacity, and effectiveness in diffusion-based separation processes. These membranes also exhibit enhanced resistance to fouling and mechanical stress, ensuring reproducibility and sustained performance in DD applications [44].

Recent advances in channel-engineered ion-exchange membranes have shown that nanoscale alignment of ionic domains and the formation of selective nanochannels can significantly enhance ion mobility and selectivity [45]. These developments emphasize the growing importance of structure–transport relationships in membrane design, providing the rationale for investigating the commercial Fujifilm membranes within a quantitative optimization framework.

Donnan dialysis has been widely studied for the removal of ionic contaminants and valuable resources such as copper, fluoride, nitrate, arsenate, aluminum, gold, lithium, and phosphate from water and wastewater [36,37].

Traditional methods for heavy metal removal are often costly, time-consuming, and inefficient, particularly when dealing with large sample volumes and chemical reagents [46,47]. These approaches frequently fall short in process optimization. In contrast, modern techniques employing advanced statistical tools, such as Response Surface Methodology (RSM), offer greater efficiency and resource savings [48].

This study employs a systematic approach combining RSM and Central Composite Design (CCD) to evaluate the removal efficiency of copper and nickel from aqueous solutions. The CCD-RSM framework enables simultaneous analysis of multiple independent variables, their individual and interactive effects, and potential synergies. This methodology addresses the limitations of traditional experimental designs by reducing the number of required trials while maintaining statistical robustness [49,50,51].

CCD is widely recognized for its effectiveness in optimizing complex systems involving three to six experimental factors. Originally developed by Box and Wilson, CCD utilizes a second-order polynomial model incorporating linear, quadratic, and interaction terms to describe the relationships between input variables and response outcomes. The design includes full or fractional factorial components, axial points, and center points to assess curvature and interactions comprehensively.

A pilot study was conducted to ensure the selected experimental ranges were appropriate. CCD combines heuristic exploration with higher-order modeling to achieve precise optimization across the studied factors and their interactions [52,53].

The primary objective of this research is to maximize the simultaneous removal efficiency of Cu^2+^ and Ni^2+^ ions from aqueous solutions using Donnan dialysis. This study introduces a novel integration of DD with advanced design methodologies, employing RSM and CCD to optimize copper and nickel separation. It also evaluates the performance of two Fujifilm ion-exchange membranes (Type 1 and Type 2) in relation to counter-ion selection (Na^+^ vs. H^+^), leveraging the user-friendly NemrodW software (Version 9901)for condition optimization. The findings offer valuable insights for improving industrial practices in heavy metal treatment, emphasizing both efficiency and cost-effectiveness.

## 2. Experiments

### 2.1. Reagents and Method

Analytical grade reagents were used. Copper(II) sulfate pentahydrate (CuSO_4_·5H_2_O, M = 249.68 g·mol^−1^, purity ≥ 99%, Sigma-Aldrich, St. Louis, MO, USA) and nickel(II) sulfate hexahydrate (NiSO_4_·6H_2_O, M = 262.85 g·mol^−1^, purity ≥ 99%, Sigma-Aldrich) were used as Cu^2+^ and Ni^2+^ sources. NaCl (M = 58.44 g·mol^−1^, purity ≥ 99.5%) and HCl (37% *w*/*w*) were used to prepare receiver solutions.

Masses reported correspond to preparation of 1.00 L of a 100 mg·L^−1^ metal solution (as element): CuSO_4_·5H_2_O, 0.393 g; NiSO_4_·6H_2_O, 0.448 g. Working solutions were obtained by dilution.

Metal concentrations were measured by ICP-OES(Agilent, Paris, France). Wavelengths: Cu 324.754 nm, Ni 231.604 nm. Calibration employed at least five points (e.g., 0, 1, 5, 10, 15 mg·L^−1^) and matrix-matched standards.

The cell consisted of two 25 mL compartments (feed and receiver) separated by the membrane, all of which were stirred magnetically at 300 rpm.

Feed solutions contained Cu^2+^ and Ni^2+^ ions at concentrations ranging from 10 mg/L to 100 mg/L; the receiver phase was 0.01 mol/L to 0.2 mol/L NaCl solution unless otherwise stated. Both compartments were securely sealed. During each run, samples were collected hourly from both compartments for analysis. The resulting volume reduction was taken into account during data processing and mass balance calculations. The mass balance was established to ensure the conservation of the solute mass (Cu^2+^ or Ni^2+^) between the two compartments at any given time. The total mass at time t can be expressed as:

M_total_ (t) = m_f_ (t) + m_r_(t)

where m_f_(t) and m_r_(t) represent the total masses of Cu^2+^ or Ni^2+^ in the feed and receiver compartments, respectively.

### 2.2. Membranes

Two commercial cation-exchange membranes (CEM Type I and CEM Type II), both with homogeneous structures, were supplied by Fujifilm (Fujifilm Manufacturing Europe B.V., Tilburg, The Netherlands) [54]. These membranes feature a three-dimensional polyolefin inert fiber-based structure [55] and are fabricated via electrospinning [56].

Each membrane was cut into circular coupons (effective area = 28.3 cm^2^, diameter = 6 cm).

Prior to use, all Fujifilm cation-exchange membranes were equilibrated in 0.1 mol/L NaCl solution for 24 h at room temperature. After equilibration, membranes were rinsed with deionized water and gently blotted before assembly in the dialysis cell.

After each run, both compartments and the membrane were thoroughly rinsed with deionized water and reconditioned with 0.1 mol/L NaCl for 24 h before reuse. After five cycles, a new membrane was used, or whenever surface inspections indicated fouling or integrity loss. Every run was performed in triplicate to ensure reproducibility.

Table 1 summarizes the key characteristics of the swollen membranes, including type, structure, fixed groups, ion-exchange capacities (wet and dry), thickness (dry and wet), conductivity, density, water content, permeability, and transport number. These parameters are critical for evaluating membrane performance in Donnan dialysis applications.

### 2.3. Donnan Dialysis

The Donnan dialysis (DD) process offers several environmentally friendly advantages, including operational simplicity, low installation and maintenance costs, and minimal energy consumption [62]. A key benefit of DD over other membrane technologies is that it does not require an applied electrical potential or pressure gradient, thereby reducing energy demands [63].

**Figure 1:**
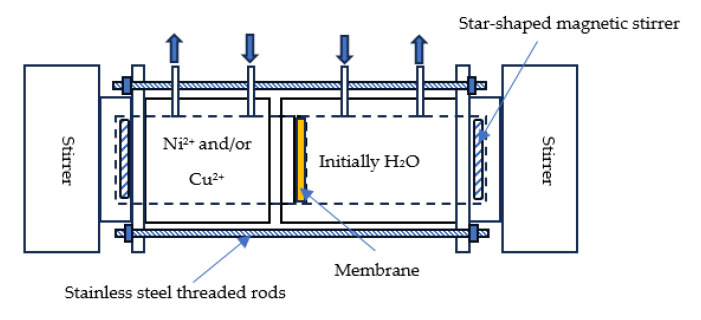
Donnan Dialysis Cell.

All DD experiments were conducted using a laboratory-scale dialysis cell composed of two detachable compartments made of polymethylmethacrylate (PMMA), commonly known as plexiglass. As illustrated in Figure 1, the cell consists of four parts connected by stainless steel threaded rods. Each compartment includes threaded openings fitted with stuffing boxes to securely hold the membrane in place. The membrane is clamped between the compartments to ensure a tight, leak-proof seal during operation [64].

Each end of the cell features a central cavity housing a star-shaped magnetic stirrer, driven by an external rotating magnetic field to provide continuous agitation. This design ensures optimal flow conditions and enhances mass transfer during the dialysis process.

The removal efficiencies of copper (Y_1_%) and nickel (Y_2_%) were calculated using the following Equation (1):

(1) $$Y_{1};Y_{2}\%=\frac{C_{0}-C_{e}}{C_{0}}\times 100$$

where C_e_ is the copper and nickel equilibrium concentration (mg/L) and C_0_ is the initial concentration of copper or nickel (mg/L).

### 2.4. Optimisation Software

The experimental design for Response Surface Methodology (RSM) was based on a Central Composite Design (CCD) matrix to determine the optimal conditions for copper and nickel ion removal. The experimental data were analyzed using NEMRODW software (Version 9901) [65], which facilitated regression analysis and estimation of the coefficients for the corresponding regression equations.

In addition to regression modeling, Analysis of Variance (ANOVA) was employed to evaluate the statistical significance of the model and its parameters. ANOVA was essential for identifying the influence of each factor and validating the adequacy of the regression model. The analysis confirmed that the fitted equations accurately represented the experimental data and could be used for reliable prediction and optimization [66,67].

## 3. Results and Discussion

### 3.1. Characterization of Membranes by FTIR

FTIR spectra of Fujifilm Type I and Type II membranes were obtained using a Tensor 27 Platinum ATR spectrometer (Bruker, Billerica, MA, USA), operated with Opus^®^ software. This fully automated system enables high-resolution spectral analysis across the near-, mid-, and far-infrared regions and is suitable for both solid and liquid samples. Its versatility makes it ideal for detailed investigations of molecular structures and the identification of functional groups in membrane materials.

**Figure 2:**
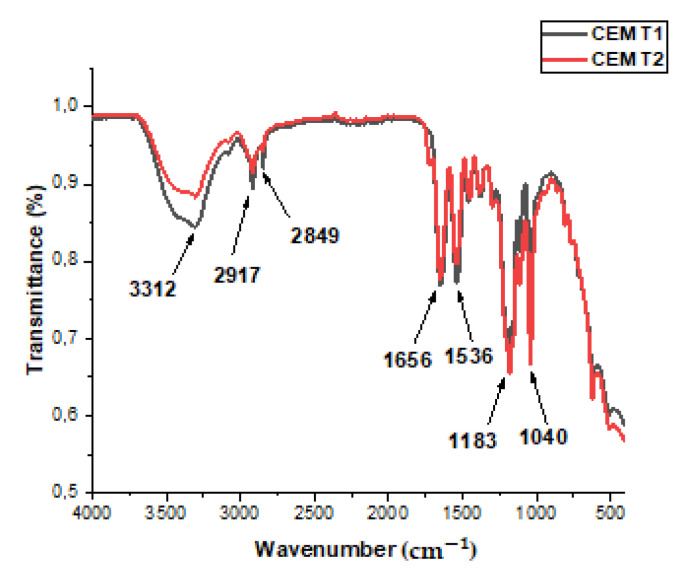
IR spectrum of CEM Fujifilm Type I and Type II.

Figure 2 presents the IR spectra of the cation-exchange membranes (CEMs), which exhibit distinct absorption bands corresponding to specific functional groups. The broad band around 3312 cm^−1^ is attributed to hydrogen-bonded N–H stretching vibrations from amine and imine groups. Peaks at 2917 cm^−1^ and 2849 cm^−1^ correspond to C–H stretching vibrations in –CH_2_– and –CH_3_ groups, respectively. The absorption band at 1656 cm^−1^ is assigned to the stretching vibration of carbonyl (C=O) groups, while the peak at 1536 cm^−1^ indicates C=N stretching, confirming the presence of imine functionalities. Additionally, the peak near 1040 cm^−1^ is indicative of sulfonic acid (–SO_3_H) groups, which are essential for ion-exchange functionality [54].

Both membrane types contain amine, imine, carbonyl, and sulfonic acid groups, which contribute to their chemical stability and ion-exchange capabilities. However, differences in the intensity of certain IR absorption bands suggest variations in the concentration of these functional groups, which may influence membrane performance in terms of ion-exchange capacity, chemical resistance, and hydrophilicity.

These assumptions are supported by analytical data. For instance, CEM Type I membranes exhibit a higher volume fraction of electroneutral solution in intergel spaces compared to Type II, likely due to the open-ended pores formed by reinforcing fabric filaments. This structural distinction explains the observed differences in transport properties between the two membrane types [44].

Furthermore, the ion-exchange capacity of CEM Type I membranes was measured at approximately 2.02 mmol/g (dry), whereas Type II membranes exhibited a slightly lower value of 1.81 mmol/g. These differences reflect variations in functional group density, which account for the superior ion-exchange performance of Type I membranes.

In summary, although both membranes share similar functional groups essential for ion exchange, their distinct chemical compositions and structural features result in different performance characteristics.

The transport of Cu^2+^ and Ni^2+^ across the Fujifilm membranes primarily follows a Donnan–diffusion mechanism. Fixed sulfonic acid groups (-SO_3_^−^) within the polymer matrix establish an internal electrostatic potential (the Donnan potential) that governs ion exchange and selectivity. Counter-ions (Cu^2+^, Ni^2+^) migrate through the membrane to maintain electroneutrality, while co-ions are effectively excluded. The overall ion flux depends on the interplay among hydration energy, charge density, and membrane morphology.

Type I membranes, which exhibit higher ion-exchange capacity (IEC) and water uptake, possess more interconnected hydrated channels that facilitate ion transport. In contrast, Type II membranes have a denser polymer network and lower IEC, leading to reduced diffusivity and lower ion flux. The slightly lower diffusion rate of Cu^2+^ compared with Ni^2+^ is offset by stronger electrostatic affinity toward sulfonic sites, explaining the higher observed Cu^2+^ selectivity in the experimental data.

### 3.2. Characterization of Membranes by SEM

**Figure 3:**
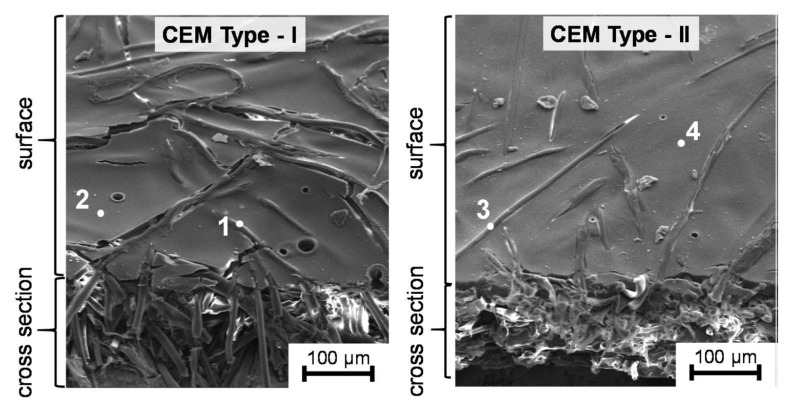
SEM images of the surface and cross-sections of cation-exchange IEM [44].

Figure 3 presents SEM micrographs of the surface and cross-section of the two Fujifilm cation-exchange membranes (CEM Type I and Type II). Clear morphological differences can be observed between the two materials.

CEM Type I exhibits a dense, compact, and uniform structure, with well-oriented polymer fibrils and a smooth surface morphology.

Where point (1) denotes a defect-free smooth polymer domain and point (2) highlights small surface depressions or micro-voids.

The cross-sectional view reveals a fairly homogeneous internal texture consisting of tightly packed domains and continuous channels, indicative of a fine organization of polymer chains and higher structural integrity. These characteristics suggest a higher ion-exchange capacity, greater water content, and enhanced intrinsic permeability of the membrane. Together, these features promote ion mobility and enable more efficient transport during Donnan dialysis.

In contrast, CEM Type II displays a coarse and rough outer morphology, with a more heterogeneous structure in which micro-voids and irregular fibrillar patterns are apparent. Where point (3) corresponds to a rough, discontinuous surface region, whereas point (4) denotes elongated fibrillar features and micro-voids. The cross-sectional view reveals a looser and more porous subsurface, indicative of weaker mechanical cohesion and reduced water uptake. This structural difference explains the lower permeability and diminished ion-exchange performance of Type II compared with Type I.

Overall, the SEM analysis confirms that CEM Type I possess greater compactness and a more continuous microstructure, resulting in faster and more selective transport of Cu^2+^ and Ni^2+^ ions through well-hydrated pathways [44].

### 3.3. Preliminary Study

#### 3.3.1. FT Influence of the Counter-Ion Nature

A preliminary study was conducted to identify the most suitable counter-ion and the optimal membrane for the intended application. The selection of the counter-ion was primarily guided by several criteria: high ionic mobility to promote efficient ion transport, environmental safety through non-toxicity, low cost for economic feasibility, and chemical inertness to prevent adverse interactions—ensuring that the counter-ion would not interfere with the treatment process. A comprehensive literature review identified both H^+^ and Na^+^ as fulfilling these requirements, positioning them as the most suitable candidates for the counter-ion role [68]. These ions are known for their high mobility, widespread availability, cost-effectiveness, and minimal reactivity with typical solution components, making them ideal for use in various membrane-based separation processes.

**Figure 4:**
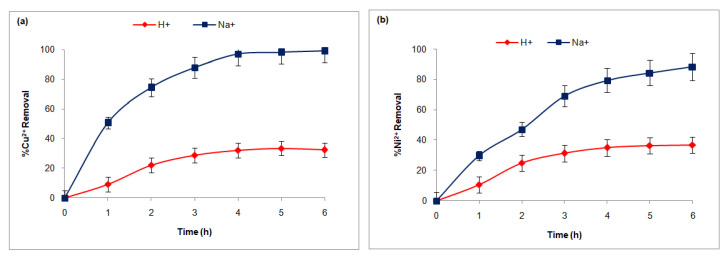
Concentration variation of copper (**a**) and nickel (**b**) with the effect of the counter-ion nature.

Figure 4a,b illustrates the temporal progression of Cu^2+^ and Ni^2+^ concentrations in the receiving compartment. The data reveals a continuous and gradual increase in the concentrations of both metals, indicating a steady transfer or removal process. Notably, the figures highlight that Na^+^ ions facilitate higher rates of Cu^2+^ and Ni^2+^ elimination compared to H^+^ ions.

The observed data suggest that Na^+^ ions enhance the removal efficiency of copper and nickel, potentially due to their inherent chemical properties and the ion-exchange mechanisms involved. NaCl was selected as the receiving compartment medium for several reasons: it is widely available, cost-effective, non-corrosive, and suitable for large-scale applications [69]. Additionally, NaCl exhibits good ionic mobility, supporting efficient ion exchange and diffusion processes. Crucially, NaCl does not cause significant pH variations in the treated solutions, ensuring the stability of the treatment process and minimizing the risk of undesired side reactions associated with acidic or basic solutions.

Recent research [70] highlights the beneficial role of sodium chloride in enhancing the removal of Cu^2+^ and Ni^2+^ ions from aqueous media. NaCl not only improves the adsorption efficiency of various sorbents but also promotes selective extraction mechanisms. For example, the addition of NaCl to solutions containing Cu^2+^ and Ni^2+^ has been shown to enhance the adsorption of these ions by up to 63.42% and 133.49%, respectively, when using specific adsorbents. This enhancement is attributed to the ionic strength and salting-out effects induced by NaCl, which favor the aggregation of metal ions onto adsorbent surfaces [71,72].

Moreover, NaCl serves as an effective medium in solvent extraction processes. In systems separating cobalt from mixtures with Cu^2+^, Ni^2+^, and Mn^2+^ ions, the presence of NaCl in the raffinate solution has been shown to facilitate the formation of cobalt complexes, thereby enabling its selective extraction [73].

The superior effectiveness of Na^+^ ions in promoting the elimination of Cu^2+^ and Ni^2+^ can be attributed to their role in increasing the ionic strength of the solution. This increase enhances the electrostatic interactions between metal ions and adsorbents or extraction agents, thereby improving removal efficiency. Additionally, NaCl’s non-corrosive nature and cost-effectiveness make it an optimal choice for large-scale applications in water treatment and metal recovery processes.

In summary, incorporating NaCl into treatment processes targeting Cu^2+^ and Ni^2+^ ions provide notable benefits, including improved adsorption capacity, facilitated selective extraction, and practical applicability for industrial and environmental heavy metal remediation.

#### 3.3.2. Choice of the Membrane

**Figure 5:**
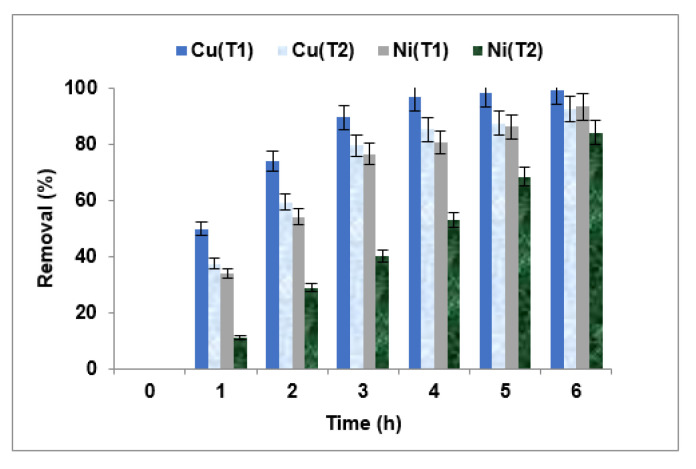
Selecting of membrane types.

Donnan dialysis was carried out using a sodium ion concentration of 0.1 mol/L and initial copper and nickel concentrations of 100 mg/L. Figure 5 presents the removal efficiencies of copper and nickel from the same compartment, with both metals tested simultaneously using two different cation-exchange membranes: Fujifilm Type I and Fujifilm Type II, after 6 h of operation.

The figure illustrates the concurrent removal of Cu^2+^ and Ni^2+^ using the two membranes. The presence of both metal ions in the same compartment appears to enhance their mutual elimination, likely due to synergistic transport dynamics.

According to the results, the Fujifilm Type I membrane exhibits superior performance in removing both copper and nickel. Specifically, the removal rates achieved with Type I were 99.13% for Cu^2+^ and 93.34% for Ni^2+^. In contrast, the Type II membrane demonstrated lower removal efficiencies: 92.47% for Cu^2+^ and 84.12% for Ni^2+^.

The enhanced performance of the Type I membrane can be attributed to its favorable physicochemical properties. It possesses a high permeability of 15 mL/bar·m^2^·h, an ion-exchange capacity of 1.43 meq/g, a transport number of 0.985, and a water content of 29% [44]. These characteristics collectively contribute to the membrane’s ability to efficiently transport copper and nickel ions from the feed compartment to the receiver compartment [74,75].

In contrast, the Type II membrane exhibits comparatively lower performance due to its reduced ion-exchange capacity (1.35 meq/g), lower water content (25%), and significantly lower permeability (3.5 mL/bar·m^2^·h). These limitations hinder the membrane’s capacity to facilitate effective ion transport, resulting in diminished removal efficiencies for both metal ions.

In conclusion, the results clearly demonstrate that the Fujifilm Type I membrane outperforms Type II in the simultaneous removal of copper and nickel ions from aqueous solutions via Donnan dialysis. Based on these findings, the Fujifilm Type I membrane was selected for the subsequent phases of the study.

#### 3.3.3. Effect of Counterion Concentration in the Receptor Compartment

This study investigates the influence of counter-ion concentrations in the receiver compartment on the removal of copper and nickel ions from the feed compartment, assessed independently. The counter-ion plays a critical role in determining the efficiency of metal ion removal through the membrane during the Donnan Dialysis (DD) process, as it governs the ion-exchange mechanism in which metal ions (Cu^2+^ and Ni^2+^) are exchanged with compensating ions from the receiver compartment.

Sodium ions were selected as the counter-ion in this study due to their favorable transport properties, particularly their high mobility, which can significantly enhance ion exchange during Donnan Dialysis. Their widespread application in membrane-based separation processes is attributed to their ability to promote the migration of target metal ions from the feed solution toward the receiving phase.

To examine the effect of Na^+^ concentration on the removal efficiency of Cu^2+^ and Ni^2+^ ions, experiments were conducted with Na^+^ concentrations in the receiver compartment ranging from 0.01 mol/L to 0.2 mol/L. In all cases, the initial concentrations of Cu^2+^ and Ni^2+^ in the feed compartment were maintained at 100 mg/L. The performance of the Fujifilm Type I cation-exchange membrane was evaluated over a 6-h period for each set of conditions. This study, therefore, focuses on the impact of Na^+^ concentrations in governing transport efficiency, thereby offering useful guidance for optimizing operational parameters in metal separation systems using Donnan Dialysis.

**Figure 6:**
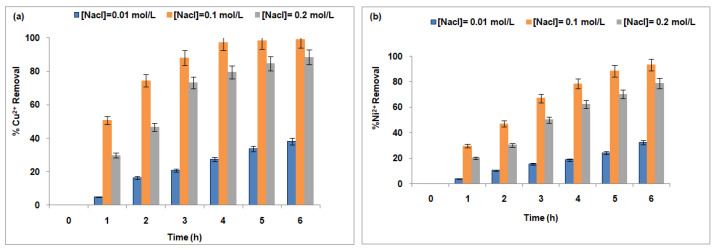
Variation of Cu^2+^ (**a**) and Ni^2+^ (**b**) removal rates with Na^+^ concentrations in the receiver.

Figure 6 shows the variation in Cu^2+^ (a) and Ni^2+^ (b) removal rates with Na^+^ concentrations in the receiver compartment. At an initial Na^+^ concentration of 0.01 mol/L, the removal efficiencies are relatively low: 38.2% for Cu^2+^ and 32.47% for Ni^2+^. This suggests that at low Na^+^ concentrations, the membrane’s ion-exchange capacity is not sufficiently activated to efficiently remove these metal ions.

However, a significant increase in removal efficiency is observed as the Na^+^ concentration rises. At 0.1 mol/L Na^+^, the removal percentages for Cu^2+^and Ni^2+^ reach 99.13% and 93.34%, respectively. This pronounced enhancement highlights a strong dependence of the membrane’s ion-exchange performance on the concentration of the counter-ion. The elevated Na^+^ levels likely intensify the driving force for ion exchange, thereby improving the membrane’s capacity to displace and extract metal ions from the feed solution.

The influence of competing cations, such as Na^+^, on metal ion exchange processes has been previously reported. A study examining the influence of various cations and anions on ion exchange noted that the presence of Na^+^ could affect the removal efficiency of metals like Cu^2+^ and Ni^2+^. However, the impact varies depending on the specific ion-exchange material and the process conditions [76].

Despite the increase in removal efficiency at 0.1 mol/L Na^+^, a subsequent decline is observed when the Na^+^ concentration reaches 0.2 mol/L. The removal rates for Cu^2+^ and Ni^2+^ decrease to 88.31% and 78.83%, respectively. This reduction suggests that the membrane’s performance may reach a saturation point or threshold, beyond which excessive Na^+^ concentrations interfere with or compete against the exchange of target metal ions, thereby diminishing overall removal capacity.

This decline could be attributed to the membrane’s ion-exchange sites becoming saturated, membrane fouling, or changes in the electrochemical gradients that govern ion transport [77]. As the Na^+^ concentration increases, these gradients may shift in a way that impairs the membrane’s ability to effectively separate Cu^2+^ and Ni^2+^ ions.

Previous investigations, including those by [39,40], have reported that high concentrations of sodium ions can negatively influence the removal efficiency of certain metal cations. These findings are supported by observed reductions in the extraction rates of divalent ions such as calcium and magnesium under elevated sodium conditions.

In conclusion, while Na^+^ concentration plays a crucial role in enhancing the performance of the Fujifilm Type I membrane in ion removal, there appears to be an optimal Na^+^ concentration (0.1 mol/L) beyond which the removal efficiency begins to decline. This highlights the importance of carefully optimizing Na^+^ levels to ensure maximum membrane performance in Donnan Dialysis applications.

Here the initial concentrations of Cu^2+^ and Ni^2+^ in the feed compartment are the concentrations of copper and nickel in natural waters can vary significantly depending on factors such as geographic location, nearby industrial activities, the presence of mineral deposits, and prevailing environmental conditions. In response to these variations, copper and nickel concentrations were independently studied in the feed compartment to evaluate their distinct effects.

For this investigation, three concentrations of copper and nickel were selected: 10 mg/L, 100 mg/L, and 200 mg/L. This range was chosen to evaluate the impact of different metal concentrations on the system. Throughout the experiments, the concentration of sodium ions in the receiving compartment was held constant at 0.1 mol/L to eliminate variability due to sodium and isolate the effects of the target metals.

**Figure 7:**
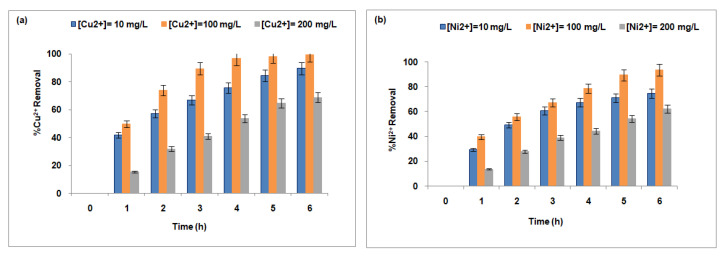
Effect of Cu^2+^ (**a**) and Ni^2+^ (**b**) concentration in the feed compartment on their removal rates, Na^+^ concentration in the receiver was equal to 0.1 mol/L.

Figure 7 illustrates the effect of Cu^2+^ (a) and Ni^2+^ (b) concentrations in the feed compartment on their respective removal rates. The performance of the Fujifilm Type I membrane demonstrates a progressive increase in removal efficiency as the concentrations of copper and nickel increase from 10 mg/L to 100 mg/L.

At a low concentration of 10 mg/L, the membrane achieves removal efficiencies of approximately 89.7% for copper and 74.87% for nickel. This initial difference suggests a greater affinity of the membrane for copper ions, which may be attributed to more favorable transport kinetics, stronger interactions with membrane exchange sites, or enhanced electrochemical potential gradients [78]. Under these conditions, ion removal primarily occurs through passive diffusion and limited ion exchange, mechanisms that appear to favor copper ion transport.

When the feed concentration increases to 100 mg/L, both ions show a marked improvement in removal efficiency, reaching 99.4% for copper and 93.52% for nickel. This increase is largely driven by the steeper concentration gradient across the membrane, which acts as a stronger driving force for mass transfer. The intensified gradient accelerates metal ion diffusion toward the receiving compartment and promotes cross-diffusion phenomena, particularly between sodium and copper ions, further enhancing transport efficiency. For nickel, the stronger gradient also supports more effective ion exchange with sodium, resulting in improved removal, although efficiency remains lower compared to copper.

Overall, the concentrations of copper and nickel ions play a critical role in determining the effectiveness of membrane-based separation systems. Optimal removal is typically observed at moderate concentrations, where diffusion and sodium-driven ion exchange dominate the transport mechanism. As metal ions are progressively removed, the system exhibits increased specific removal rates, suggesting enhanced efficiency under these conditions. This trend indicates that at reduced concentrations, fundamental transport forces—such as concentration gradients and ion-exchange dynamics—operate more effectively, contributing to higher overall removal performance.

At higher concentrations of 200 mg/L, a decline in membrane performance is noted for both ions. This decrease is mainly due to the saturation of ion-exchange sites and the onset of membrane fouling, which collectively reduce the membrane’s capacity to retain and transport ions. These factors lead to diminished retention and increased leakage of ions into the permeate [79]. While increased concentrations initially boost ion transport by strengthening the chemical potential gradient, the system eventually reaches a threshold where ion-exchange capacity is overwhelmed, causing performance deterioration marked by blocked active sites and reduced permeability, consistent with prior studies [77,80].

Across the entire concentration range tested, copper consistently shows superior removal compared to nickel. This difference likely stems from copper’s higher ionic mobility, stronger affinity for the membrane surface, and more effective cross-diffusion dynamics. Conversely, nickel exhibits slower transport kinetics, particularly at lower concentrations. Although effective removal of both ions is achieved at elevated concentrations, the membrane demonstrates clear selectivity favoring copper, especially pronounced at lower feed levels.

In summary, while both copper and nickel ions follow comparable transport pathways through the Fujifilm Type I membrane, copper consistently exhibits superior removal efficiency across the concentration range tested. An increase in feed concentration from 10 mg/L to 100 mg/L enhances the removal of both metals, primarily due to the intensification of the concentration gradient, which drives diffusion and ion exchange processes. However, copper benefits more significantly from cross-diffusion and a more apparent electrochemical gradient, which further facilitates its transport through the membrane. At higher concentrations (>200 mg/L), the removal efficiency for both ions declines due to ion-exchange site saturation and membrane fouling, which limit overall system performance. Despite these limitations, copper consistently remains more effectively removed than nickel under all conditions investigated.

### 3.4. Optimization by Response Surface Methodology Using Central Composite Design (CCD)

Response Surface Methodology (RSM) is a systematic approach that uses mathematical and statistical techniques for experimental design, analysis, model development, and optimization [81,82]. It helps investigate and improve complex processes by creating empirical models (Equation (2)) that link process variables to expected responses, facilitating process refinement with fewer experimental trials [83]. RSM is widely used in industries for optimizing new products and processes and improving existing ones, ensuring that quality specifications are met while enhancing performance, cost-efficiency, and overall product quality [84].

(2) $$Y=b_{0}+\Sigma_{i=1}^{k}b_{i}X_{i}+\Sigma_{i=1}^{k}b_{\mathrm{ii}}X_{i}^{2}+\Sigma_{i=1}^{k-1}\Sigma_{j=i+1}^{k}b_{\mathrm{ij}}X_{i}X_{j}+\varepsilon$$

where Y is the predicted response, b_0_ is the intercept, b_i_ is the linear coefficient, b_i_ is the quadratic coefficient, and b_i_ is the two-factor interaction coefficient; X_i_ are the coded independent variables, and ε is the residual error. This formulation now fully agrees with the fitted models presented in Equations (4), (6) and (7).

The Central Composite Design (CCD) is an experimental technique employed to model and optimize processes by examining the linear and quadratic effects of input variables [85]. It facilitates the development of an empirical second-order polynomial model, which is essential for understanding the interactions between factors and their impact on the process outcome [86].

The design includes a first-order 2^k^ factorial component, supplemented by 2^k^ axial points to investigate curvature effects, and center runs to identify nonlinearities. Most of the information regarding the response system and the evaluation of factor significance is derived from the first-order design. The number of experimental runs required for modeling and process optimization is determined by Equation (3), which incorporates all components of the experimental design [87]:

(3) $$N=\;2^{k}+2k+Nc$$

In the present study, the central composite design (CCD), a subset of response surface methodology (RSM), was employed to investigate the individual and interactive effects of three independent variables: (X_1_) copper concentration, (X_2_) nickel concentration, and (X_3_) sodium ion concentration, on two dependent responses: (Y_1_) copper removal efficiency (%) and (Y_2_) nickel removal efficiency (%). The factor levels for the independent variables were coded as −1.0 (Low), 0.0 (Center), and +1.0 (High).

The factor levels for the independent variables were coded as −1.0 (Low), 0.0 (Center), and +1.0 (High). The ranges and levels of the independent factors, determined through preliminary studies and used for statistical design of the experiments via CCD, are presented in Table 2.

The selected model uses a second-order polynomial equation (Equation (4)) to describe the predicted values of the responses Y:

Y= b_0_

(4) $$Y=b_{0}+b_{1}X_{1}+b_{2}X_{2}+b_{3}X_{3}-b_{11}X_{1}^{2}-b_{22}X_{2}^{2}-b_{33}X_{3}^{2}+b_{12}X_{1}X_{2}-b_{13}X_{1}X_{3}-b_{23}X_{2}X_{3}$$

where Y denotes the predicted response, b_0_is the constant of coefficient b_i_ is the linear regression coefficient, b_ij_ is the regression coefficient for two-factor interaction effects, b_ii_ represents the regression coefficient for quadratic main effects, X_i_ and X_j_ are the coded of factors [88].To validate the model, the regression coefficient (R^2^) and the percentage of absolute deviation (AED%) between experimental and predicted results were calculated using Equation (5):

(5) $$\mathrm{AED}\%\;=\;\frac{100}{N}.\left|\frac{Y_{exp}-Y_{theo}}{Y_{exp}}\right|$$

where Y_exp_ and Y_theo_ represent the experimental and theoretical values respectively, and N denotes the number of measurement points. A model was considered valid if it met at least one of the following criteria: (R^2^ > 0.8) or (AED < 10%) [89].

According to the CCD matrix (Table 3), a total of 17 runs were required, comprising 8 factorial points, 6 axial points, and 3 center points.

Using the experimental findings and the second-order polynomial equations (Equations (6) and (7)), the data were fitted to the model:

(6) $$Y1\;=\;90.026\;+\;13.014\;X_{1}\;+\;5.832\;X_{2}\;+\;1.188\;X_{3}\;-\;12.651X_{1}^{2}-\;0.899X_{2}^{2}-\;12.151\;X_{3}^{2}-10.229\;X_{1}X_{2}+5.829\;X_{1}X_{3}-1.104\;X_{2}X_{3}$$

(7) $$Y2=86.898+12.789\;X_{1}+6.475\;X_{2}+1.245\;X_{3}-10.642\;X_{1}^{2}-1.822\;X_{2}^{2}-7.422\;X_{3}^{2}-10.094\;X_{1}X_{2}+4.844\;X_{1}X_{3}-0.781\;X_{2}X_{3}$$

The regression coefficients (R^2^) were 0.999 for copper and 0.998 for nickel, with AED values of 0.279% and 0.512%, respectively—well below the 10% threshold—confirming the models’ validity (Table 4).

**Figure 8:**
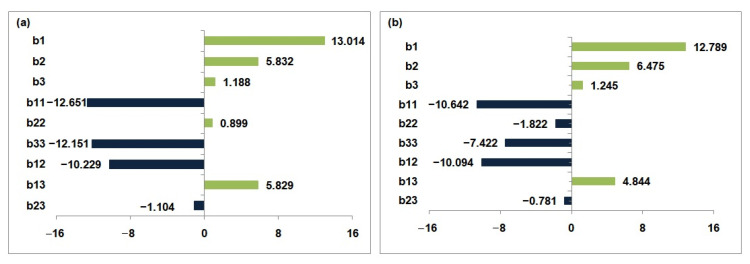
Graphical analysis of the effects of factors on the central composite design for the removal of (**a**) copper, (**b**) nickel.

The graphical analysis provides valuable insights into the overall effect of each factor, as well as itscombined influence on the response variables. Based on the coefficients derived from Equation (6) and the graphical representation in Figure 8a, the concentration of copper in the feed compartment (b_1_ = 13.014) is identified as the most significant factor influencing copper removal, underscoring its dominant role in the process. The concentration of nickel (b_2_ = 5.832) emerges as the second most influential factor, although its effect is considerably less pronounced than that of copper. In contrast, the concentration of sodium ions (b_3_ = 1.188) exerts a relatively minor influence on copper removal.

Similarly, the regression coefficients obtained from Equation (7), along with the graphical representation in Figure 8b, indicate that copper concentration in the feed compartment (b_1_ = 12.789) also exerts the most significant influence on nickel removal. This finding reinforces the dominant role of copper in governing the ion-exchange dynamics within the system. In comparison, nickel concentration (b_2_ = 6.475) emerges as the second most impactful variable, though its effect is notably less pronounced than that of copper. Sodium ion concentration (b_3_ = 1.245) demonstrates a comparatively moderate influence on nickel removal efficiency, further highlighting the relatively minor role of Na^+^ in this context.

These results collectively suggest that the concentrations of both copper and nickel are critical parameters in optimizing the Donnan Dialysis process, with copper playing a particularly pivotal role in enhancing the removal performance for both metal ions. The relatively minimal impact of Na^+^ concentration further emphasizes the need to prioritize the optimization of copper and nickel concentrations within the feed compartment to enhance overall removal efficiency.

The removal of heavy metal ions such as Cu^2+^ and Ni^2+^ is influenced by several physicochemical properties, including ionic radius, electronegativity, molecular weight, and hydration energy [90,91,92]. Cu^2+^ has a larger ionic radius (0.79 Å) than Ni^2+^ (0.69 Å), which affects its mobility through the membrane and its interaction with the membrane surface. The difference in removal efficiency between Ni^2+^ and Cu^2+^, despite their similar electronegativities, may also be attributed to the higher atomic radius of Ni^2+^ (149 pm for Ni^2+^ vs. 128 pm for Cu^2+^) [93].

Moreover, Cu^2+^ has a lower hydration energy (−2100 kJ/mol) compared to Ni^2+^ (−2105 kJ/mol), which allows for stronger interactions with the membrane matrix [94]. Additionally, Cu^2+^ has a higher molecular weight (63.556 g/mol) than Ni^2+^ (58.700 g/mol) [95,96,97], which tends to favor its removal. Differences in ionic size and molecular weight also affect ion mobility, leading to higher ion concentrations within the membrane boundary layer and consequently enhancing removal efficiency.

Factors such as hydration energy and ionic radius play a significant role in governing the interactions between ions and the membrane surface, thereby influencing the membrane’s capacity to selectively remove these ions. As reported by [98] in their adsorption study, metals with higher molecular weights are generally removed to a greater extent than those with lower molecular weights.

#### 3.4.1. Pareto Chart and Contribution

A Pareto chart is an analytical chart which combines a bar chart and a cumulative line graph to show the importance of the different contributing factors of a problem. Each factor is represented on the bar chart according to its frequency or magnitude, starting from the highest value first, and the line graph represents the factors’ cumulative contributions to ensure that the most important factors are readily identified.

**Figure 9:**
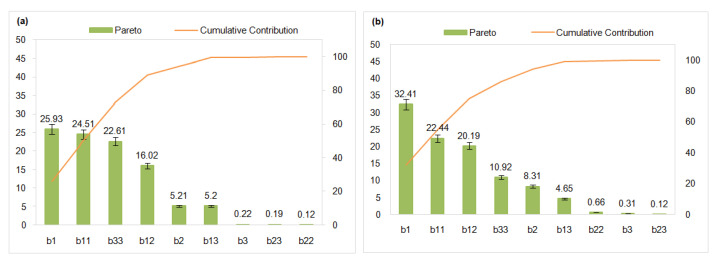
Pareto chart and cumulative contribution of the removal of concentration of (**a**) copper and (**b**) nickel.

In Figure 9a every bar corresponds to a factor or category which is ranked as copper, copper-sodium, copper-nickel, nickel, sodium, and nickel-sodium, with the height of every bar signifying one specific value or magnitude of that category and contributing copper-sodium and sodium. The line diagram depicts the cumulative contribution of each category as a running total arranged in descending order on the right *y*-axis. Out of the three, the highest figure which makes an individual contribution of 25.93% is Copper, which is then followed by copper-sodium 24.51% and copper-nickel 22.61%. This figure indicates the level of contribution measured as the copper-sodium contribution and the line reflects closing in on approximately 50% after enduring both categories. Most pertinent is that the initial few categories accounted for most value in the contribution while the remaining categories tend to offer progressively smaller amounts.

Each bar in the specific Pareto chart shown in Figure 9b depicts a category or factor, and its height shows its corresponding frequency or impact; in this case, they are copper, copper-nickel, copper-sodium, nickel, sodium, and copper-sodium, nickel-sodium, and nickel-sodium, with copper as the most significant at 32.41%. The cumulative line indicates the driven value and accounts for all the lower-level categories from 0, depicting how much of the total is measured with the added top categories. With this observation, it is clear that the first few categories on the left provide most input with value claimed to be most contributed by copper at 32.41%, while further right shifts result in a lesser value from each additional category, resulting in a flattening of the line. This chart exemplifies the Pareto principle, also known as the 80/20 rule, whereby a few left-side categories contribute extensive value, while the many switches on the right are awarded comparatively minimal input.

In summary, this Pareto chart shows that copper and copper-nickel are the most substantial categories, paying more attention to them. Every effort needs to be made to ensure that they devise solutions that will yield the most positive change as far as delicate processes are concerned.

#### 3.4.2. Analysis of Variance ANOVA

In ANOVA, a statistical technique, two types of variables are identified: control factors and response variables. The primary objective of ANOVA is to assess the influence of control factors on the response variables. The significance of each control factor in the ANOVA table is assessed using the corresponding *p*-values, where a *p*-value less than 0.05 indicatesstatistical significance [99].

Modeladequacy was validated using variance analysis (ANOVA). The obtained results are summarized in Table 5. The *p*-value represents the error. In order to identify statistically significant effects, the *p*-value (ratio of the mean square effect) and the F-ratio (mean square) were calculated. A *p*-value close to zero suggests that the data are significant. The ANOVA results indicate a *p*-value below 0.05 and an F-value greater than the critical Fishervalue F(9,7,0.05) = 3.68. Therefore, the model is statistically significant and appropriate for describing the copper and nickel removal process.

#### 3.4.3. Residual Analysis

Residual analysis and normal probability plots are essential tools for assessing the adequacy of a statistical model. Residual analysis involves examining the differences between observed and predicted values (residuals) to check for randomness, constant variance, and the absence of outliers, as well as ensuring that the residuals are normally distributed. The normal probability plot serves as a graphical method to visually determine whether the residuals conform to a normal distribution by plotting the ordered residuals against the expected quantiles of a theoretical normal distribution. If the points on the plot deviate from a straight line, it indicates non-normality, which can affect the validity of statistical tests. Together, residual analysis and normal probability plots ensure that a model meets the assumptions necessary for reliable inference [100].

**Figure 10:**
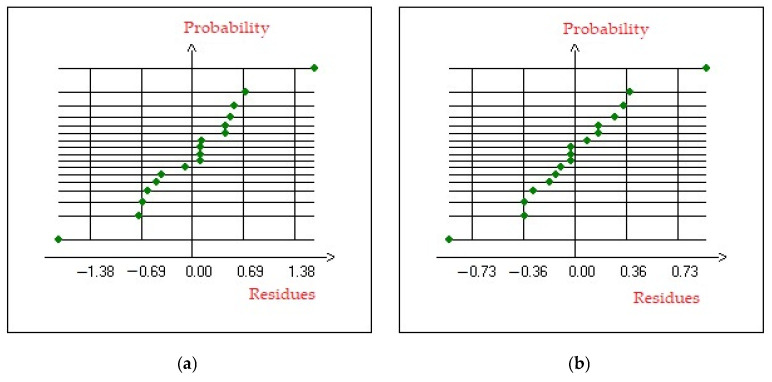
Normal probability plot of residue of (**a**) copper and (**b**) of nickel.

The residuals of the proposed model were subjected to normality assessments to verify their conformity to a Gaussian distribution, thereby confirming the statistical validity and robustness of the model. The close alignment of residuals along Henry’s line suggests the absence of significant non-linear behavior, indicating that the model faithfully represents the physicochemical transport mechanisms involved. This conclusion is further supported by the probability and normal residual plots presented in Figure 10a,b, which demonstrate that residuals fall within the expected normal distribution range, reinforcing the model’s reliability.

**Figure 11:**
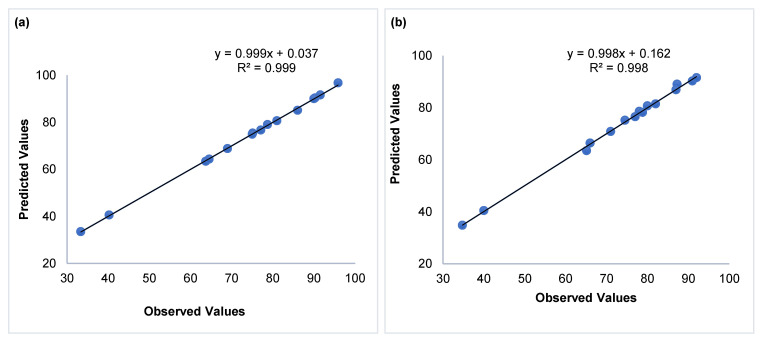
Predicted versus Observed values plot of (**a**) removal of Cu^2+^ and (**b**) removal of Ni^2+^.

Furthermore, the plots comparing observed and predicted values, shown in Figure 11a,b, exhibit a strong linear correlation, indicating that the regression model successfully captures the variability of the experimental data across the investigated parameter space. The close agreement between fitted regression equations and experimental results, coupled with high coefficients of determination (R^2^), validates the model’s applicability for both predictive simulations and process optimization in membrane-based separation systems.

### 3.5. The 3D Surface Plots and 3D Contour Plots

To comprehensively illustrate the removal behavior of copper and nickel during Donnan dialysis, contour plots were used to display lines of constant response values, showing how the efficiency changes in relation to the studied variables. These plots are valuable for identifying optimal zones where ion removal is maximized.

Complementing this, three-dimensional response surface graphs were produced to explore the interactions between pairs of variables in greater detail. Unlike contour plots, which focus on fixed response levels, 3D surfaces reveal how combinations of factors jointly affect the removal performance. This method provides a deeper understanding of how multiple parameters work together to influence the overall efficiency of copper and nickel extraction.

**Figure 12:**
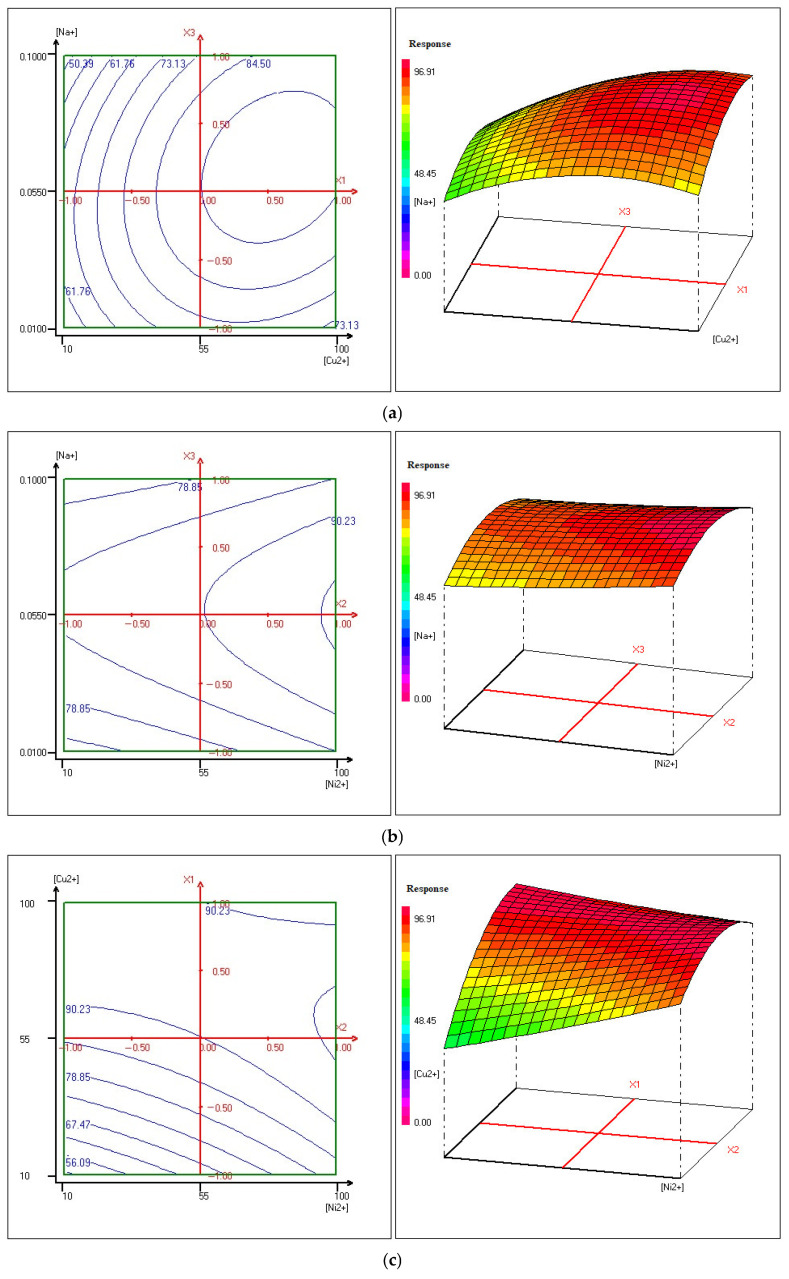
Contour plots and the corresponding three-dimensional plots of Y_Cu2+_: (**a**) Sodium concentration versus copper concentration; (**b**) Sodium concentration versus nickel concentration; (**c**) Copper concentration versus nickel concentration.

**Figure 13:**
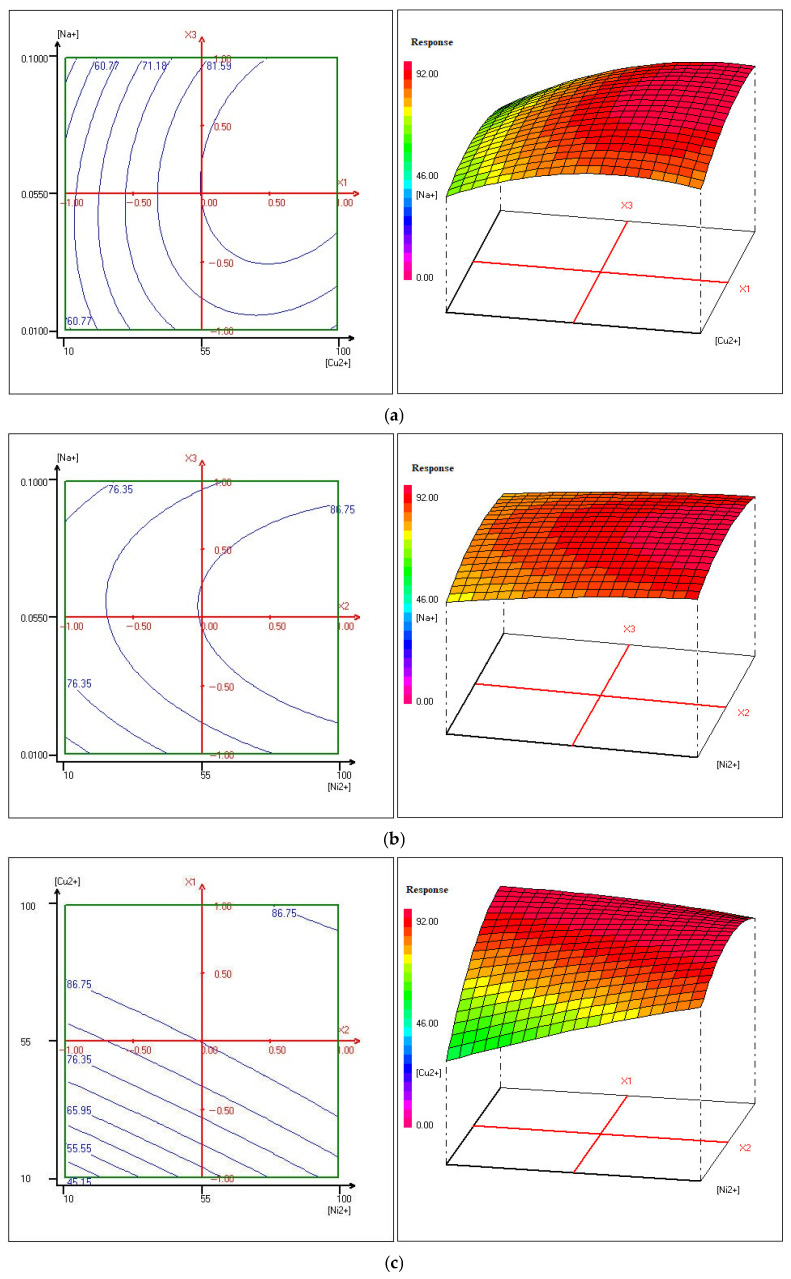
Contour plots and the corresponding three-dimensional plots of Y_Ni_^2+^: (**a**) Sodium concentration versus copper concentration; (**b**) Sodium concentration versus nickel concentration; (**c**) Copper concentration versus nickel concentration.

The 3D response surface plots, presented in Figure 12 and Figure 13, offer a more detailed visualization of these interactions, enabling a better understanding of how to optimize the removal of copper and nickel under different conditions. This integrated use of contour plots and 3D response surfaces serves as a powerful tool for optimizing the Donnan dialysis process.

This methodology is based on the principles established by [101,102] who extensively applied response surface methodology to study and optimize complex processes involving multiple variables.

The analysis of the contour plots in Figure 12a reveals that the concentrations of copper and sodium significantly influence the elimination process. When copper concentration is between 10 and 55 mg/L, the contour lines are spaced, indicating that the copper elimination rate remains relatively constant and is less sensitive to increases in copper concentration within this range. However, when the copper concentration exceeds 55 mg/L and reaches up to 100 mg/L, the contour lines show more variation, with a maximum elimination rate of 93.75% ± 0.28% observed in this range, suggesting that optimal copper elimination occurs when its concentration is maintained between 55 mg/L and 100 mg/L. Regarding sodium, when its concentration ranges from 0.01 mol/L to 0.1 mol/L, the contour lines are more widely spaced, indicating that changes in sodium concentration within this range have minimal impact on the elimination rate. Therefore, the process is less sensitive to sodium concentration variations in this range.

In summary, the highest copper elimination rate (93.75% ± 0.28%) is achieved when Cu^2+^ concentration is between 55 mg/L and 100 mg/L, and Na^+^ concentration is between 0.038 mol/L and 0.088 mol/L, representing the optimal zone for copper elimination. To achieve maximum elimination, careful regulation of Cu^2+^ and Na^+^ concentrations within these ranges is necessary, with the concentration gradient of counterions likely facilitating the exchange of two Na^+^ ions for each Cu^2+^ ion while maintaining system electroneutrality. This analysis underscores the importance of precisely controlling copper and sodium concentrations to optimize the elimination process.

Figure 12b illustrates the interaction between the concentrations of nickel and sodium in relation to the removal efficiency. The concentration ranges for Ni^2+^ (10 mg/L to 100 mg/L) and Na^+^ (0.01 mol/L to 0.1 mol/L) show that the removal percentage is relatively insensitive to changes within these ranges, as indicated by the widely spaced contour lines. The optimal conditions for maximum nickel removal occur at Ni^2+^ concentrations between 95 mg/L and 100 mg/L and Na^+^ concentrations between 0.043 mol/L and 0.066 mol/L, with the copper concentration fixed at 55 mg/L, resulting in a maximum removal rate of 91.54% ± 0.33%. This analysis highlights the ideal concentration levels for efficient nickel elimination while showing that variations within the given ranges have a minimal effect on the process.

The detailed analysis of the contour plot in Figure 12c highlights that the removal ratio is sensitive to the concentrations of both nickel and copper. The plot indicates the presence of specific optimal regions where the concentrations of these two elements fall within narrow ranges, leading to high removal efficiency.

When examining the contour curves for nickel and copper, it becomes apparent that the contour lines are widely spaced, suggesting that the removal efficiency changes gradually as the concentrations of these elements vary. In this particular range, the response to changes in nickel and copper concentrations is relatively less sensitive. This implies that small fluctuations in concentration within this zone have minimal effects on the removal percentage.

Specifically, the highest removal of copper (96.89% ± 0.25%) occurs within the concentration range of 48 mg/L to 72 mg/L, while the highest removal of nickel (91.72% ± 0.30%) is observed in the concentration range of 95 mg/L to 100 mg/L, with sodium concentration being maintained at 0.055 mol/L.

This removal behavior is influenced by the interplay of copper and nickel, which contributes to the concentration gradients of sodium ions. The movement of these ions across the system intensifies cross-ion transfers, helping maintain electroneutrality within the system.

Figure 13a illustrates the combined influence of copper and sodium concentrations on copper removal, with a constant nickel concentration of 55 mg/L. The contour lines representing different copper and sodium concentrations indicate that the copper removal rate remains relatively stable and exhibits reduced sensitivity to increases in copper concentration.

As the copper concentration increases from 55 mg/L to 100 mg/L, the copper removal rate initially increases. However, this rate begins to decrease as the sodium concentration rises from 0.07 mol/L to 0.1 mol/L.

The optimal conditions for copper removal are characterized by a maximum removal efficiency of 94.5% ± 0.27%, which occurs when copper concentrations range from 55 mg/L to 100 mg/L, and sodium concentrations are between 0.03 mol/L and 0.062 mol/L. These conditions define the ideal parameters for achieving maximum copper elimination.

It is essential to precisely control both Cu^2+^ and Na^+^ concentrations within these optimal ranges to maximize removal efficiency. The concentration gradient of counterions likely facilitates the exchange of two Na^+^ ions for each Cu^2+^ ion, thereby maintaining system electroneutrality. This highlights the critical role of controlling copper and sodium concentrations to optimize the copper removal process.

Figure 13b presents a detailed analysis of the relationship between sodium concentration and nickel removal, while maintaining a constant copper concentration of 55 mg/L. The data reveals a nuanced trend in nickel removal efficiency based on sodium concentration levels.

At lower sodium concentrations, ranging from 0.01 mol/L to 0.02 mol/L, there is a noticeable decline in the removal of nickel. This could be attributed to the slower movement of ions, particularly the Na^+^ ions, which results in less efficient exchange with nickel ions. In this range, the ion interaction is not rapid enough to promote effective removal of nickel from the solution.

However, as sodium concentration increases from 0.02 mol/L to 0.092 mol/L, a marked improvement in the removal of nickel is observed. This increase in nickel removal efficiency correlates with higher sodium concentrations, which likely facilitate a more effective ion exchange process. This trend is especially pronounced when the initial nickel concentration falls within the 54 mg/L to 100 mg/L range, with a peak removal efficiency of 92.66% ± 0.31% noted. The higher sodium concentrations likely enhance the mobility of nickel ions, thus improving their removal.

Furthermore, the contour lines in the figure become more widely spaced, indicating that the nickel removal rate stabilizes and remains relatively constant. This suggests the presence of a saturation point in the efficiency of nickel removal, beyond which increases in sodium concentration no longer significantly enhance removal efficiency.

In Figure 13c, the variation between the concentration of copper, the nickel concentration and the sodium concentration was fixed to 0.055 mol/L. The results indicate that at lower concentrations of nickel, an increase in copper concentration significantly influences the removal of nickel. This suggests that copper concentration plays a crucial role in the efficiency of nickel removal under these conditions.

However, as the concentration of nickel rises, there is a noticeable improvement in the removal of nickel itself. This indicates a positive relationship between nickel concentration and the effectiveness of nickel removal via Donnan dialysis. Specifically, the increased concentration of nickel enhances the ion exchange process, leading to more effective removal.

When analyzing the copper removal performance, it becomes clear that copper concentration also plays a significant role in the studied response. The highest copper removal, 95.87% ± 0.26%, occurs within a concentration range of 30 mg/L to 92 mg/L. In contrast, nickel removal achieves its highest efficiency, 92.62 ± 0.29%, within a concentration range of 80 mg/L to 100 mg/L, while maintaining a constant sodium concentration of 0.055 mol/L.

The observed improvement in removal efficiencies can be attributed to the increased concentration gradients of nickel and copper. These gradients facilitate enhanced flux of sodium ions from the receiver solution to the feed solution. The increased ionic flux promotes the cross-ion transfer between sodium ions and the metal ions, thus maintaining the electroneutrality of the system. This process directly contributes to the enhanced efficiency of both copper and nickel removal during the dialysis process.

The contour plots generated in this study delineate the optimal concentration ranges for three key parameters required to achieve removal rates exceeding 90%. Utilizing NEMRODW software (Version 9901) alongside the desirability function, precise optimal values were identified: 80 mg/L for copper, 100 mg/L for nickel, and 0.06 mol/L for sodium. Underthese conditions, removal efficiencies reached outstanding levels, with copper and nickel eliminated at rates of 99.67% ± 0.21% and 98.85% ± 0.34%, respectively, with (*n* = 3). These findings underscore the critical role of fine-tuning the concentrations of copper, nickel, and sodium to maximize the dialysis system’s effectiveness. The analysis highlights that meticulous adjustment and control of these variables—particularly the metal ion concentrations—are essential for optimizing ion removal and achieving superior overall process efficiency.

An additional validation experiment (Cu^2+^ = 80 mg/L, Ni^2+^ = 100 mg/L, Na^+^ = 0.06 mol/L) was conducted outside the CCD design to assess the prediction accuracy of the RSM model. The calculated removal efficiencies were confirmed experimentally (Cu^2+^ removal = 99.67 ± 0.21%, Ni^2+^ removal = 98.85 ± 0.34%), demonstrating the model’s reliability and negligible lack-of-fit. The previously reported regression metrics (R^2^ = 0.999 and 0.998; AED < 1%) were thus validated by this independent test and remain accurate as described above.

Experimental verification performed in triplicate under the RSM-optimized conditions yielded consistent results (Cu^2+^ = 99.67 ± 0.21%, Ni^2+^ = 98.85 ± 0.34%), confirming the reproducibility of the process. Over five consecutive operating cycles, the Fujifilm Type 1 membranes retained more than 98% of their initial performance, demonstrating strong mechanical and chemical stability. Moreover, the Donnan dialysis setup operated under mild conditions, with commercially available membranes and without any external energy input—features that suggest the feasibility of scaling up the process through stacked multi-cell membrane configurations. These aspects highlight the robust practicality of the optimized model for potential deployment in the treatment of industrial wastewaters containing heavy metals.

Donnan dialysis using commercial Fujifilm cation-exchange membranes achieved comparable or superior removal efficiencies (>98% for Cu and >96% for Ni under optimized conditions) while operating without external energy input and under ambient conditions. In contrast, nanocomposite and graphene-based membranes, as well as NF/UF approaches, have also reported high Cu rejection (typically >90%) but generally require applied pressure, chemical complexation, or additional pre/post-treatment steps [62].

Donnan and diffusion dialysis processes are increasingly recognized for their selective ion transport and low energy requirements, with several reviews emphasizing their potential for metal recovery and separation without the need for hydraulic or electrical driving forces [62]. Highly effective heavy metal removal has also been reported for various nanocomposite and graphene-derivative membranes. For instance, in bench-scale tests, layered graphene oxide and MOF/GO nanocomposite membranes have achieved Cu removal efficiencies around 90–95%, while enhancing antifouling resistance and selectivity when incorporated into polymer matrices [103]. Catalytic nanocomposite membranes combining MoS_2_/GO within chitosan or other functional matrices have similarly shown high simultaneous removal of dyes and metal ions, demonstrating that membrane modification is an effective route to improve capacity and selectivity [104].

Ultrafiltration coupled with complexation (e.g., sodium polyacrylate + UF) and nanofiltration have also been successfully applied for Cu^2+^ and Ni^2+^ separation, with NF achieving >90% Cu rejection in mining wastewater tests, though at the cost of high operating pressures and chemical pretreatment requirements [105].

Compared with these pressure-driven or chemically assisted alternatives, the optimized Donnan dialysis process operating under ambient conditions achieved very high removal efficiencies (Cu ≈ 99.7%, Ni ≈ 98.9%) with excellent model predictability (R^2^ > 0.998). This confirms that Donnan dialysis using commercial Fujifilm cation-exchange membranes is a competitive and energy-efficient method for heavy metal removal and recovery, particularly when operational simplicity, low energy demand, and modular scalability are required. Nevertheless, nanocomposite and NF/UF technologies may offer advantages in terms of flux, fouling resistance, or integrated catalytic performance, depending on feed composition and site-specific constraints. Therefore, the selection of the most appropriate technology should balance throughput, energy availability, pretreatment needs, and the intended end-use of the recovered metals [62].

## 4. Conclusions

This study offers novel and in-depth insights into the use of cation-exchange membranes, particularly Fujifilm, for the removal of copper and nickel ions through Donnan Dialysis. FT-IR analysis confirmed the presence of key functional groups responsible for ion exchange, while the role of sodium ions as effective counter-ions was clearly demonstrated. Notably, Na^+^ at a concentration of 0.1 mol/L significantly enhanced metal ion removal; however, further increases led to diminished performance due to ion-exchange site saturation and potential membrane fouling. Among the membranes studied, Fujifilm Type I consistently outperformed its counterpart, achieving up to 99.13% removal of Cu^2+^ and 93.34% of Ni^2+^. This superior performance is attributed to its greater ion-exchange capacity, higher hydrophilicity, and stronger affinity for heavy metal ions.

The integration of Central Composite Design (CCD) with Response Surface Methodology (RSM) enabled precise modeling of the process, identifying key operational parameters and their interactions. The resulting models demonstrated excellent predictive strength (R^2^ > 0.998; AED < 1%), with copper concentration emerging as the most influential factor, while sodium and nickel concentrations played important but secondary roles in the removal process.

The elimination efficiencies ranging between (93.75 ± 0.28% and 96.89 ± 0.25%) correspond to local maxima within the RSM design space.

However, the global optimum predicted by the desirability function was achieved at Cu^2+^ = 80 mg/L, Ni^2+^ = 100 mg/L, and Na^+^ = 0.06 mol/L, giving model-predicted efficiencies of 99.67 ± 0.21% (Cu^2+^) and 98.85 ± 0.34% (Ni^2+^). These values were experimentally validated in triplicate, yielding 99.45 ± 0.18% (Cu^2+^) and 98.71 ± 0.27% (Ni^2+^), confirming the model’s robustness and predictive accuracy.

The distinction between local and global optima emphasizes that while contour plots reveal process sensitivity zones, the global optimum defined by RSM desirability (≈99%) represents the statistically validated operational target for maximum removal efficiency.

The Donnan dialysis system functions under mild conditions without the need for external energy and achieves high efficiencies in the removal and recovery of Cu^2+^ and Ni^2+^ using commercially available cation-exchange membranes. Although these membranes exhibited satisfactory preliminary operational stability and reproducibility, further investigations are required to evaluate long-term durability, resistance to fouling, and the cost implications associated with NaCl consumption and handling of the receiver solution. Within these limitations, the method exhibits great promise for the effective and sustainable removal and recovery of heavy metals from aqueous systems.Future techno-economic and lifecycle analyses aiming at expanding Donnan dialysis systems for the treatment of industrial wastewater will have a strong basis thanks to these findings.

## Figures and Tables

**Table 1:** Characteristics of the membranes studied.

Membranes	Fujifilm Type 1	Fujifilm Type 2	Reference
Type	Homogeneous	Homogeneous	[44]
Structure property	Aliphatic polyamide	Aliphatic polyamide	[44]
Fixed groups	-SO_3_^−^	-SO_3_^−^	[44]
Exchange capacity (mmol/g wet)	1.43 ± 0.05	1.35 ± 0.05	[44,57,58]
Exchange capacity (mmol/g dry)	2.02	1.81	[44]
Thickness of Air-Dried Membrane (µm)	120 ± 5	165 ± 5	[55,59]
Thickness of Wet Membrane (µm)	140 ± 10	180 ± 10	[55]
Conductivity ($\bar{\kappa }$, mS/cm)	6.1	2.8	[44]
Density (g/cm^3^ wet)	1.15	1.13	[44]
Water Content (g H_2_O/g wet, %)	29 ± 5	25 ± 2	[44]
Permeability (mL/bar hm^2^)	15	3.5	[55,60]
Transport number	0.985	0.996	[44,61]

**Table 2:** Range and the levels of removal factors.

Coded	Factors	Symbol	Range and Levels		
			Low	Center	High
X_1_	Concentration of Cu^2+^ (mg/L)	[Cu^2+^]	−1 10	055	1 100
X_2_	Concentration of Ni^2+^ (mg/L)	[Ni^2+^]	−1 10	055	1 100
X_3_	Concentration of Na^+^ (mol/L)	[Na^+^]	−1 0.01	00.055	1 0.1

**Table 3:** Matrix of composite and results.

No. Exp	X_1_	X_2_	X_3_	[Cu^2+^]	[Ni^2+^]	[Na^+^]	Y_1exp_(%)	Y_1Cal_(%)	Y_2exp_(%)	Y_2Cal_(%)
1	−1	−1	−1	10	10	0.0100	40.23	40.586	40.00	40.472
2	1	−1	−1	100	10	0.0100	75.12	75.414	77.00	76.550
3	−1	1	−1	10	100	0.0100	75.00	74.915	74.50	75.172
4	1	1	−1	100	100	0.0100	69.00	68.828	71.00	70.875
5	−1	−1	1	10	10	0.1000	33.33	33.512	34.75	34.837
6	1	−1	1	100	10	0.1000	91.56	91.655	91.00	90.290
7	−1	1	1	10	100	0.1000	63.71	63.426	66.00	66.412
8	1	1	1	100	100	0.1000	81.00	80.654	82.00	81.490
9	−1	0	0	10	55	0.0550	64.53	64.361	65.11	63.467
10	1	0	0	100	55	0.0550	90.26	90.389	87.25	89.045
11	0	−1	0	55	10	0.0550	86.02	85.093	78.00	78.601
12	0	1	0	55	100	0.0550	95.87	96.757	92.00	91.551
13	0	0	−1	55	55	0.0100	77.08	76.687	78.80	78.231
14	0	0	1	55	55	0.1000	78.71	79.063	80.00	80.721
15	0	0	0	55	55	0.0550	90.00	90.026	87.00	86.898
16	0	0	0	55	55	0.0550	90.00	90.026	87.00	86.898
17	0	0	0	55	55	0.0550	90.00	90.026	87.00	86.898

**Table 4:** Model constants, *p* values, and R^2^ for the copper and nickel removal.

	Y_1_(%)		Y_2_(%)	
	Coefficients	*p*-Values	Coefficients	*p*-Values
b_0_	90.026	0.0000	86.898	0.0000
b_1_	13.014	0.0000	12.789	0.0000
b_2_	5.832	0.0000	6.475	0.0000
b_3_	1.188	0.0004	1.245	0.0434
b_11_	−12.651	0.0000	−10.642	0.0000
b_22_	0.899	0.0422	−1.822	0.0373
b_33_	−12.151	0.0000	−7.422	0.0000
b_12_	−10.229	0.0000	−10.094	0.0000
b_13_	5.829	0.0000	4.844	0.0000
b_23_	−1.104	0.0012	−0.781	0.0074
R^2^	0.999		0.998	

**Table 5:** Analysis of variance.

Source Model	Degree of Freedom	Sum of Square	Mean of Square	F-Value	F_tableFischer_ (α = 5%)	*p*-Value
Cu^2+^						
Regression	9	4993.31	554.812	1575.6401	3.68	0.000<
Residual	7	2.4649	0.352118			
Total	16	4995.77				
Ni^2+^						
Regression	9	4258.75	473.194	360.3067	3.68	0.000<
Residual	7	9.19317	1.31331			
Total	16	4267.94				

## Data Availability

The data presented in this study are available on request from the corresponding author.
